# Comparison of measured residential black carbon levels outdoors and indoors with fixed-site monitoring data and with dispersion modelling

**DOI:** 10.1007/s11356-020-12134-8

**Published:** 2020-12-19

**Authors:** Olena Gruzieva, Antonios Georgelis, Niklas Andersson, Tom Bellander, Christer Johansson, Anne-Sophie Merritt

**Affiliations:** 1 Institute of Environmental Medicine, Karolinska Institutet, Nobels väg 13, SE-17177 Stockholm, Sweden; 2 Centre for Occupational and Environmental Medicine, Region Stockholm, Stockholm, Sweden; 3 Department of Environmental Science, Stockholm University, Stockholm, Sweden; 4 Environment and Health Administration, SLB-analys, Stockholm, Sweden

**Keywords:** Black carbon, Routine monitoring, Dispersion modelling, Indoor/outdoor ratio

## Abstract

Epidemiologic studies on health effects of air pollution usually rely on time-series of ambient monitoring data or on spatially modelled levels. Little is known how well these estimate residential outdoor and indoor levels. We investigated the agreement of measured residential black carbon (BC) levels outdoors and indoors with fixed-site monitoring data and with levels calculated using a Gaussian dispersion model. One-week residential outdoor and indoor BC measurements were conducted for 15 families living in central Stockholm. Time-series from urban background and street-level monitors were compared to these measurements. The observed weekly concentrations were also standardized to reflect annual averages, using urban background levels, and compared spatially to long-term levels as estimated by dispersion modelling. Weekly average outdoor BC level was 472 ng/m^3^ (range 261–797 ng/m^3^). The corresponding fixed-site urban background and street levels were 313 and 1039 ng/m^3^, respectively. Urban background variation explained 50% of the temporal variation in residential outdoor levels averaged over 24 h. Modelled residential long-term outdoor levels were on average comparable with the standardized measured home outdoor levels, and explained 49% of the spatial variability. The median indoor/outdoor ratio across all addresses was 0.79, with no difference between day and night time. Common exposure estimation approaches in the epidemiology of health effects related to BC displayed high validity for residencies in central Stockholm. Urban background monitored levels explained half of the outdoor day-to-day variability at residential addresses. Long-term dispersion modelling explained half of the spatial differences in outdoor levels. Indoor BC concentrations tended to be somewhat lower than outdoor levels.

**Supplementary Information:**

The online version contains supplementary material available at 10.1007/s11356-020-12134-8.

## Introduction

Epidemiological studies of air pollution and adverse health effects mainly rely on temporal or spatial estimates of outdoor levels. The data for temporal analysis are generally obtained from fixed monitoring, assuming that these time-series are representative of the population exposure. Various monitoring studies have suggested that in urban areas, high variability of air pollution may occur over small distances (Nikolova et al. 2011; Bell et al. 2007). Hence, air pollution data from a single monitoring station can be representative of a rather small surrounding area. Such exposure measurements are usually affected by the monitor’s location, and may not adequately capture the spatial variability for pollutants in areas with local sources which in turn may result in an underestimation of the inter- and intra-variability of personal exposure within the study population (Dionisio et al. 2013; Dias and Tchepel 2018). Long-term studies often use residential outdoor estimates as the indicator of exposure, assuming these spatial contrasts to be good predictors of individual exposure contrasts. However, dissimilarities in exposure-response associations for different exposure assessment approaches highlight the importance of detailed exposure assessment (Dionisio et al. 2016; Brokamp et al. 2019; Dias and Tchepel 2018).

Black carbon (BC) is a constituent of PM_2.5_ fine particulate matter (particles with an aerodynamic diameter less than 2.5 μm), mainly originating from the incomplete combustion of fossil and biomass fuels, and suspected to greatly contribute to particle toxicity in urban air. BC is thus considered as an indicator to assess the adverse effects of particulates on human health where combustion sources dominate, showing more robust associations with morbidity, mortality, and life expectancy than PM_2.5_ (WHO. 2013). The majority of previous studies examining the relationship between measured pollutant concentrations and predicted by spatial models have focused on regulated air pollutants (e.g. nitrogen oxides, particulate matter), whereas data on BC are scarce. Several studies have reported generally moderate to low agreement between modelled and measured personal BC exposure estimates (Montagne et al. 2013; Nieuwenhuijsen et al. 2015; Nethery et al. 2008). These studies utilized exposure data modelled by means of land use regression (LUR); meanwhile, our study is the first to investigate how well dispersion models predict measured BC levels at residential addresses.

In addition to the significant temporal and spatial variability of outdoor air pollution levels, existing evidence has demonstrated that the indoor environment plays a significant role in personal exposure to air pollution (Zou et al. 2009). In developed countries, people usually spend a vast majority of their time indoors (Monn 2001), suggesting that some of the observed health effects from air pollution are due to outdoor air pollution that infiltrates to the indoor environment. In the past decade, a number of studies investigated the indoor/outdoor (I/O) ratio of various air pollutants; however, data on BC are still limited (Wichmann et al. 2010; Gotschi et al. 2002).

The aims of the present study were to quantify the following: (1) the temporal agreement between measured outdoor concentrations of BC at the home address, and at ambient air quality monitoring stations, (2) the spatial agreement of these measurements with calculated BC levels from a dispersion model, and 3) the indoor/outdoor ratio of measured residential BC levels.

## Materials and methods

### Study population

The present study was based on an ongoing cohort EMIL (Etiological Mechanisms of air pollution effects in the Infant Lung), with an overall aim to elucidate biological mechanisms behind the induction of adverse effects by air pollution during early life. In brief, the EMIL cohort comprises about 100 newborn children from Stockholm that had been recruited after identification in a population register. Data on residential characteristics, socio-economic, and lifestyle factors were collected via parental questionnaires when children were 6 months, 1 year, and 2 years of age, along with clinical examinations at the same ages.

At the time when a child turned 1 year, parents received an invitation to participate in personal measurements of black carbon. Inclusion criteria were as follows: families where both parents lived at the same address, and that one parent was working outside the home while the other was on parental leave at home with a child. Sampling took place during April 2016–June 2017. The study was approved by the Ethics Committee of Karolinska Institutet. The parents of all participating families provided written informed consent. All participants of this project received financial compensation for their contribution.

## Black carbon exposure assessment

### Residential measurements

**Fig. 1:**
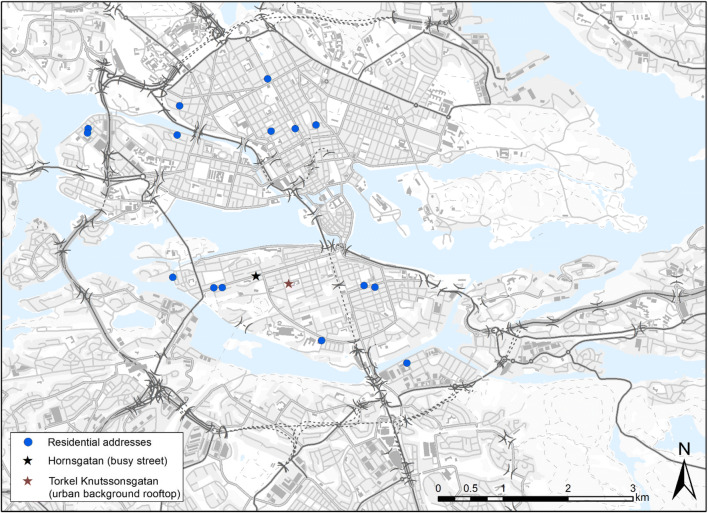
Geographical location of residential addresses of the participants in the EMIL study as well as air quality monitoring stations in Stockholm. Each circle represents residential individual address; stars represent air quality monitoring stations

Black carbon was measured in 15 families at their homes (Fig. 1) continuously for seven consecutive days, with a battery-operated MicroAeth Model AE51 (Aethlabs, San Francisco, CA, USA) on a 5-min time resolution. The instrument works by drawing air through a fiber filter at a constant flow rate (150 ml/min), resulting in BC aerosol accumulation on the filter, which is analyzed optically in real time (Merritt et al. 2019). The measured concentration is given in nanograms BC per cubic meter (ng/m^3^) of air. Before each use, the instruments were checked to ensure that they worked properly as indicated by the manufacturer. The filter was replaced every 2 days to prevent saturation and subsequent measurement bias. For the outdoor residential measurements, the aethalometer was kept indoors with a conductive sampling tube going outdoors through a window opening. In addition, both family members (the working parent and the one on parental leave) were also supplied with BC personal samplers and were instructed to carry it with them at all times with the inlet positioned in the breathing zone. When both parents were at home, the personal aethalometers were placed next to each other in the living room, and these data along with information on time-activity patterns from personal diaries were then used to obtain outdoor/indoor ratios.

During the measurement period, all study participants filled in a time-activity diary with each day divided in time intervals during which they had to provide information about their activities and locations (indoors or outdoors, time in traffic), as well as specific activities that could influence BC levels, such as smoking and stove use. In order to estimate indoor BC levels, we retrieved measurements from a personal sampler of the parent being on parental leave during the times he/she was at home (as indicated in the diary). Those measurements were then matched by time with equivalent data from the stationary device sampling the outdoor air. The ratio was calculated based on 1-h indoor and outdoor averages.

### Fixed-site monitoring and dispersion modelling

Urban background (rooftop), as well as street levels, were monitored with regular fixed-site monitors by the urban air quality monitoring network of the Environment and Health Administration, SLB, Stockholm (Krecl et al. 2011). The measurement sites were as follows: Torkel Knutssonsgatan (urban background), and Hornsgatan (busy street), both in the south part of central Stockholm (Södermalm; Fig. 1), as well as Aspvreten (rural background). The corresponding urban, rural, and street BC levels were retrieved for each home address starting and ending at the same time as the measurements at home.

We also estimated outdoor residential BC levels based on air quality dispersion modelling, using emission data and weather data, described in detail elsewhere (Segersson et al. 2017). The modelling system is part of the Airviro Air Quality Management System (http://airviro.com) and has provided exposure estimates for several epidemiological studies and health impact assessment studies (Gruzieva et al. 2012; Olsson et al. 2015; Ljungman et al. 2019). Briefly, home addresses of families were geocoded and the concentrations of BC were calculated using a Gaussian dispersion model using real-time meteorological data (as 1-h, 24-h, and 1-week averages) or using climatology (to obtain annual averages). Meteorological data (wind speed and direction, temperature gradient, and horizontal and vertical wind fluctuations) were obtained from measurements in a 50-m-high mast in southern Stockholm. A diagnostic wind model was applied in order to obtain data on wind, stability, and turbulence parameters in the whole domain. The wind model assumes that small-scale winds can be seen as a local adaptation of large-scale winds (free winds) due to local fluxes of heat and momentum from the sea or earth surface. Any non-linear interaction between the scales is neglected. It is also assumed that the adaptation process is very fast and that horizontal processes can be described by non-linear equations while the vertical processes can be parameterized as linear functions. The large-scale winds as well as vertical fluxes of momentum and temperature were estimated from profile measurements in the mast located in the southern part of the city. Topography and land use data for the Danard model are given by 100-m resolution. Since the topography of Greater Stockholm is relatively smooth, without dominating ridges or valleys, the free wind can be assumed to be horizontally uniform in the whole domain. The dispersion calculations of road traffic emissions were performed on a fixed grid with a grid size of 100 × 100 meters. Individual buildings and street canyons are not resolved but treated using a roughness parameter. In an open area, the calculation height is 2 m above ground level. Over a city, the simulation will reflect the concentrations at roof-top-level height. The Gaussian dispersion model was used to calculate concentrations due to local emissions. The contribution to the concentrations from emissions outside of the domain, for example concentrations from long-range transport of BC, was taken from the rural monitoring station Aspvreten located 70 km to the south-west of Stockholm (Jönsson et al. 2013). Annual outdoor residential BC levels were modelled using climatology, which consist of 360 different meteorological situations based on 15 years of meteorological measurements. The validity of using climatology with 360 cases, rather than all 8736 h of a calendar year, has been shown in Segersson et al. (Segersson et al. 2017) and Eneroth et al. (Eneroth et al. 2006).

### Statistical analyses

Measurement data were checked for missing values and anomalies (e.g. nonphysical data such as negative concentrations, impossibly high concentrations, or significant variation from the specified flow rates) as well as any other errors (e.g. missing data due to instrument malfunction). Measurements recorded at the time of filter change along with one preceding and following record (6.71%) or an error code (2.39%) were thus excluded. Negative measurements were included in the analysis, because a temporary false decrease in measured absorption is offset in the next observation(s) (McBean and Rovers 1998; Wallace 2005). Cleaned 5-min data were then averaged to yield mean 1-h, 24-h, and 1-week values. Averages were considered valid if ≥ 75% of the 5-min values within each exposure window were available. In addition, we used concurrent and annual BC averages measured at the urban background station to standardize the 1-week averages of BC measured at home to reflect annual averages (Hoek et al. 2002). Week averaged residential outdoor BC levels were standardized according to the following equation: week averaged measured residential outdoor BC levels − week averaged measured outdoor BC at urban background monitor + annual measured outdoor BC at urban background monitor.

The relationship between averaged measured and modelled BC concentration was investigated by means of Pearson correlation test, as well as in linear regression analysis. STATA Release 13.1 (StataCorp, College Station, Tex) was used for database management and statistical analyses.

## Results

### Description of study population and BC exposure levels

A total of 15 families have conducted indoor and outdoor BC measurements at home. All homes were located in multi-storey buildings within the center of Stockholm. Seven of these 15 apartments were based in older houses built before the year 1940, while four were in houses built after 2005 (Table S1). Half of the houses used mainly natural ventilation, and the vast majority used kitchen fans. The use of natural gas for cooking was very uncommon, as was tobacco smoking (only 2 persons reported occasional smoking, i.e. < 1 cigarette/day). Table 1 summarizes the descriptive statistics of outdoor measured and modelled levels of BC at home addresses of study participants, as well as BC levels measured at fixed monitoring stations. The 7-day average measured residential outdoor BC exposure was 472 ng/m^3^ (ranging 261–797 ng/m^3^). The measured residential weekly average outdoor BC concentrations were higher than the average urban background levels and considerably lower than measured at the street-level site. The modelled 1-week outdoor BC levels were generally similar to the measured levels, corresponding to an average level of 446 ng/m^3^ (ranging 236–735 ng/m^3^) (Table 1). When comparing annual averages, the modelled levels were similar to the standardized residential measurements, 599 and 549 ng/m^3^, respectively.

**Table 1:** Black carbon levels averaged per 1-, 24-h, and 1-week measurement period (ng/m^3^)

	*n*	Mean	SD	Median	Min–max
A) 1-h average					
Measured at home address	2038	449	358	367	25–3910
Modelled for home address	2038	455	365	367	21–4992
Measured at continuous monitor:					
• Urban background	2038	313	202	269	26–2220
• Rural background	1564	129	116	85	7–728
• Street level	2038	1006	769	778	49–5451
B) 24-h average					
Measured at home address	70	467	207	444	160–1115
Modelled for home address	70	479	238	445	162–1310
Measured at continuous monitor:					
• Urban background	70	317	133	338	97–833
• Rural background	55	137	96	110	23–421
• Street level	70	998	392	1066	321–1894
C) 1-week average					
Measured at home address	11	472	139	446	261–797
Modelled for home address	11	446	172	404	236–735
Measured at continuous monitor:					
• Urban background	11	313	66	330	218–434
• Rural background	8	131	51	120	79–228
• Street level	11	1039	140	1028	797–1188
D) 1-year average					
Measured at home address	11	549	127	565	351–811
Modelled for home address	11	599	159	598	355–867
Measured at continuous monitor:					
• Urban background	11	390			
•Rural background	11	192			

Descriptive statistics over the distribution of BC levels presented in Table 1 is based on complete case observations, i.e. when both measured and modelled BC levels within the same time intervals are non-missing. Noteworthy, these concentrations were generally comparable with the ones including all available observations (Table S2). 

### Associations of measured outdoor BC concentrations measured at fixed monitoring stations as well as modelled

The correlation matrix between measured and modelled BC outdoor levels averaged through different time periods is presented in Table S3. The correlation of measured and modelled BC levels appeared to be highest for annual averages (*r* = 0.70), while the correlation of measured and urban background BC levels appeared to be highest for short-term averages (*r* = 0.61 and 0.71, for 1- and 24-h averages, respectively).

In a univariate regression analysis, BC levels measured at the urban background fixed monitor explained 38.1%, 50.5%, and 17.4% of variation in measured 1-h, 24-h, and weekly averages, respectively (Table 2). Furthermore, street-level BC concentrations measured at the fixed monitor explained variation of observed outdoor residential BC levels to a less extent.

**Table 2:** Relationship of measured outdoor black carbon concentrations (ng/m^3^) with modelled, as well as measured at fixed monitoring stations

	Measured at home address			
		*β*	95% CI	*R*^2^
A) Averaged per 1 h (*N* = 15)				
Modelled at home address	Constant	300	278–323	9.7
	*β*	0.3	0.3–0.3	
Street-level monitor	Constant	294	270–317	9.9
	*β*	0.2	0.1–0.2	
Urban background monitor	Constant	109	87–132	38.0
	*β*	1	1–1	
B) Averaged per 24 h (*N* = 15)				
Modelled at home address	Constant	337	231–443	7.0
	*β*	0.2	0.04–0.4	
Street-level monitor	Constant	249	127–370	15.1
	*β*	0.2	0.1–0.3	
Urban background monitor	Constant	115	24–207	50.5
	*β*	1.1	0.8–1.4	
C) Averaged per 1 week (*N* = 11)				
Modelled at home address	Constant	450	182–717	0.2
	*β*	0.03	− 0.5–0.6	
Street-level monitor	Constant	401	− 321–1122	0.4
	*β*	0.1	− 0.6–0.8	
Urban background monitor	Constant	197	− 264–658	17.4
	*β*	0.9	− 0.6–2.3	
D) Averaged per 1 year (*N* = 11)				
Modelled at home address	Constant	-189	− 762.5–384	48.9
	*β*	1.4	0.31–2.4	

*Constant*, regression intercept; *β*, the regression coefficient; *R*^*2*^, coefficient of determination (%)

Modelled annual BC levels explained 48.9% of variation of the annual average levels derived from the residential outdoor measurements. For the shorter averaging periods, modelled residential outdoor BC levels explained considerably less: 9.7%, 7.0%, and 0.2% of the variation in observed residential outdoor levels averaged over 1 h, 24 h, and 1 week, respectively. Most of the modelled concentrations were within a factor of 2 of the measured outdoor residential concentrations as shown in the scatter plots in Fig. S1. However, there was a substantial variation in the explained variance of the modelled outdoor residential BC concentrations for the 15 different residential sites, as can be seen in Fig. S2. The model explained between 48 and 1.6% of the measured variation in hourly mean outdoor levels. For the daily mean concentrations, the model explained between 6.5 and 83% of the variability in the observed concentrations.

### Indoor/outdoor (I/O) BC ratio

The distribution of measured indoor and outdoor BC levels along with indoor/outdoor ratios was estimated on 1 h basis. The median I/O ratio across all addresses was 0.79, with no difference between day and night time (Table 3).

**Table 3:** Aggregated summary statistics for BC concentrations averaged over 1 h (ng/m^3^) indoors, outdoors, as well as indoor/outdoor ratios at 14 home addresses in Stockholm

	*n*	Mean	SD	Median	Min–max
Indoor, total	1092	318	355	204	22–3485
Day	484	379	418	248	22–3485
Night	608	269	286	172	26–2510
Outdoor, total	1092	389	377	280	25–3910
Day	484	467	457	343	39–3910
Night	608	326	284	242	25–2552
I/O ratio, total	1092	0.9	0.6	0.8	0.1–8.8
Day	484	0.9	0.7	0.8	0.2–8.8
Night	608	0.9	0.6	0.8	0.1–6.2

One address was excluded due to missing of indoor BC measurements

*n*, number of BC measurements averaged over 1 h. Day time (06:00–21:59); night time (22:00–05:59)

## Discussion

The present study investigated the agreement of residential black carbon levels outdoors and indoors measured in homes in central Stockholm, with fixed-site monitoring data and with dispersion modelling. We also observed that long-term dispersion modelling can predict differences in residential outdoor BC levels. Furthermore, we observed no difference in median I/O ratio during day and night time.

An earlier study from Stockholm, based on an intensive field campaign measuring BC concentrations across multiple locations, reported high hour-to-hour and day-to-day variability of BC concentrations observed at the kerbside sites (Krecl et al. 2011). Concentrations of BC between urban sites were moderately correlated even for daily averages (*R* < 0.70), combined with highly heterogeneously distributed concentrations with coefficients of divergence > 0.30 even at short spatial scales of few kilometers. The results of the present study demonstrated that street-level BC concentrations measured at a single fixed-site monitor explained much less of the variation in the observed BC levels at homes than did urban background levels, which may be at least partly due to high variability of street BC levels as shown by Krecl et al. (Krecl et al. 2011). The variability in the concentrations at kerbside street canyon sites is highly dependent on the local traffic at the site. Street width, height of buildings, and direction of street also contribute to this variability. This is in contrast to the urban background site where the concentrations are representative for sites not located in street canyons or close to busy roads. The measurements at residential locations were in most cases along streets with relatively little traffic or located on middle or top floors along more densely streets.

Our study is the first to quantify the agreement of measured BC levels with those modelled using dispersion modelling approach. Several previous studies have investigated how well measured outdoor BC levels at residential address agree with the modelled by means of LUR modelling. In Helsinki, Utrecht, and Barcelona, six 96-h outdoor and indoor measurements spread over three seasons were performed in 15 volunteers from each city (Montagne et al. 2013). The authors reported that BC LUR models developed in the framework of the European Study of Cohorts for Air Pollution Effects (ESCAPE) (Eeftens et al. 2012) explained 57%, 75%, and 33% of measured residential level variability in these areas, respectively. Our estimate of 49% explained variability for long-term outdoor levels is slightly lower than that for Helsinki area. In another study from Barcelona, hourly temporally adjusted LUR modelled estimates for the home and school addresses showed moderate correlation (*r* = 0.59) with measured personal black carbon levels at home and school over a measurement period of 2 days (Nieuwenhuijsen et al. 2015). In the HEAPS (Health Effects of Air Pollution in Antwerp Schools) study from Belgium, the calculation of daily BC concentrations using seasonal LUR models was evaluated using measurements on 42 residential locations, resulting in a correlation of 0.86 (Dons et al. 2014). In the same study, the correlation between daily concentrations measured at those 42 sites and concentrations measured at the reference monitor was 0.80, which is higher than in our study. While in our study the dispersion model better predicted outdoor residential BC concentrations standardized to an annual average, it performed worse at shorter time periods. This may at least partly be attributed to the influence of meteorological factors, which is much more important for short-term variations. Dispersion models are more uncertain in predicting hourly mean values compared to annual mean levels, where the latter is mainly dependent on the accuracy of the information on annual emissions. In addition, our earlier study comparing dispersion model results and measurements verifies that the calculated total annual concentration is well captured with this modelling methodology (see Segersson et al. (2017) for detailed discussion regarding model uncertainties). Further, we observed substantial variation in the explained variance of the modelled outdoor residential BC concentrations for the 15 different residential sites. This variation in the model’s ability to predict hourly mean BC concentrations could be due to a number of factors such as non-representativity of the point measurements for modelled 100 × 100 m gridded average concentrations, impacts of local sources not included in the emission inventory, errors of the model, or the meteorological data used by the model.

In the present study, outdoor BC levels were generally higher than indoor levels with the median ratio of the average of indoor and outdoor BC levels of 0.79. These results are in line with earlier studies utilizing measurements at homes in Stockholm (median soot I/O ratio (0.84) (Wichmann et al. 2010), as well as at non-smoking homes in Helsinki (0.79) (Koistinen et al. 2004). In another study conducted at homes in Athens, Basle, Helsinki, and Prague that were exposed to environmental tobacco smoke (ETS), the median I/O soot ratios were 0.90, 0.98, 0.91, and 1.04, respectively (Gotschi et al. 2002). Studies conducted outside Europe have more often investigated the I/O ratios for PM_2.5_ than for black carbon as summarized in a recent review (Leung 2015). For example, I/O ratios for PM_2.5_ ranged from 0.6 to 4.7 in studies from the USA (Martuzevicius et al. 2008; Polidori et al. 2007). Huang et al. demonstrated that the average I/O ratio of PM_2.5_ concentration was 0.93 near roadside areas of Guangzhou, China (Huang et al. 2007), and Massey and coworkers measured an I/O ratio of 0.98 near roadside areas of Agra, India (Massey et al. 2009). The indoor levels in our study are unlikely to be affected by the presence of indoor sources such as ETS and gas appliances as those appeared to be very uncommon (the vast majority of apartments were supplied with electric stove). The median I/O ratio was slightly higher during night time. These results, however, should be interpreted with caution due to limited sample size.

Availability of concurrent measured BC data both indoor and outdoor in a sample of families residing at various locations of Stockholm city provided a unique possibility to investigate the agreement between different exposure metrics, as well as the indoor/outdoor ratio of measured residential BC levels at addresses representing different levels of traffic-related air pollution. Although this kind of study can be burdensome for the subjects, we had good compliance from the participating families.

However, this study has some limitations. Given the labor-intensive fieldwork, the sample size was limited, which has reduced the predictive accuracy. Also, the sample population was composed of high SES non-smoking individuals living in the center of Stockholm, and the observed concentrations and relations may not be representative of all individuals in the city and hence does not reflect potential inequalities in exposure. We used output from a diagnostic wind model as input to our dispersion model; therefore, part of the discrepancy between the modelled and observed concentrations can be explained by erroneous meteorological data. It is, however, unlikely that wind direction could have affected average weekly and annual levels substantially, but for hourly mean values, wind speed and wind direction may be important for sites affected by local traffic. Finally, we performed measurements during 1 week in each family, thus representing only one season. Previous studies, including those conducted within the same geographical area, demonstrated pronounced seasonal variations in the concentrations of BC, with the highest concentrations during autumn-winter season and lowest in spring-summer (Dons et al. 2014; Olstrup et al. 2019). Measurements for longer time periods than 1 week could be considered when possible or, alternatively, conducting a second sampling campaign in a different season to have a better representation of variations in meteorological conditions and in emission sources throughout the year. Future research should further investigate the relationship between measured and modelled residential air pollution exposures and those estimated from ambient monitoring networks in larger samples, also evaluating the importance of time-activity patterns of study subjects for the estimated residential exposure levels.

## Conclusion

In conclusion, we found that short-term residential levels of outdoor levels of black carbon could be predicted by concurrent ambient urban monitoring concentrations, in particular as a 24-h average. Thus, our results provide support for using BC concentrations measured at urban background monitoring stations to predict daily variations of outdoor BC levels at residential addresses in central city areas similar to Stockholm. Furthermore, BC exposure levels modelled with dispersion modelling can be used as surrogates of population exposures in long-term studies based on spatial differences. Also, outdoor BC levels in the present study were comparable with indoor levels.

## Supplementary Information

ESM 1 (DOCX 272 kb)

## Data Availability

The datasets used and/or analyzed during the current study are available from the corresponding author on reasonable request. Supplementary information is available at Environmental Science and Pollution Research’s website.
