# Supplementary material for: Comparison of measured residential black carbon levels outdoors and indoors with fixed-site monitoring data and with dispersion modelling
Source: Environ Sci Pollut Res Int. 2020 Dec 19;28(13):16264–71. doi: 10.1007/s11356-020-12134-8 (PMC7969542; doi:10.1007/s11356-020-12134-8)
Supplement: Supplementary file 1 — (DOCX 272 kb) [file 11356_2020_12134_MOESM1_ESM.docx]

**Appendix.**

**Gruzieva et al.**

**Table S1. Characteristics of the homes of participants in the EMIL study (N=15)**

| **Characteristics** | **Frequency (N)** |
| --- | --- |
| Year the house was built:   - Before 1940 | 7 |
| - 1940-1975 | 3 |
| - 1976-2005 | 1 |
| - After 2005 | 4 |
|  |  |
| Ventilation system: |  |
| - Mechanical | 4 |
| - Natural | 8 |
| - Not known | 3 |
| Use of kitchen fan:   - No - Yes, rarely used - Yes, almost daily | 1  4  10 |
| Daily use of natural gas for cooking | 2 |

**Table S2. Black carbon levels averaged per 1-, 24-hour and 1 week measurement period including all available observations (ng/m^3^).**

|  | **n** | **Mean** | **SD** | **Median** | **Min-Max** |
| --- | --- | --- | --- | --- | --- |
|  |  |  |  |  |  |
|  | **A) 1 hour average** | | |  |  |
| Measured at home address | 2186 | 436.2 | 354 | 353 | 15 - 3910 |
| Modelled for home address | 2491 | 440 | 361 | 350 | 21 - 4992 |
| Measured at continuous monitor: |  |  |  |  |  |
| •         Urban background | 2330 | 310 | 204 | 262 | 26 - 2220 |
| •         Street level | 2455 | 992 | 751 | 777 | 49 - 5451 |
|  |  |  |  |  |  |
|  | **B) 24 hours average** | | |  |  |
| Measured at home address | 77 | 449 | 207 | 401 | 158 - 1115 |
| Modelled for home address | 91 | 448 | 234 | 377 | 116 - 1310 |
| Measured at continuous monitor: |  |  |  |  |  |
| •         Urban background | 84 | 313 | 130 | 320 | 97 - 833 |
| •         Street level | 91 | 966 | 382 | 981 | 275 - 1894 |
|  |  |  |  |  |  |
|  | **C) 1 week average** | | |  |  |
| Measured at home address | 12 | 464 | 136 | 428 | 261 - 797 |
| Modelled for home address | 15 | 438 | 169 | 401 | 236 - 735 |
| Measured at continuous monitor: |  |  |  |  |  |
| •         Urban background | 13 | 312 | 68 | 330 | 218 - 434 |
| •         Street level | 15 | 997 | 141 | 959 | 778 - 1188 |
|  |  |  |  |  |  |
|  | **D) 1 year average** | | |  |  |
| Measured at home address | 11 | 549 | 127 | 565 | 351 - 811 |
| Modelled for home address | 11 | 599 | 159 | 598 | 355 - 867 |
| Measured at continuous monitor: |  |  |  |  |  |
| •         Urban background | 15 | 390 |  |  |  |

**Table S3. Correlation matrix of the measured and modelled black carbon levels averaged per 1-, 24-hour and 1 week.**

|  | **1 hour average** | | | | **24-hour average** | | | | **1 week average** | | | | **1 year** |  |
| --- | --- | --- | --- | --- | --- | --- | --- | --- | --- | --- | --- | --- | --- | --- |
|  | Measured at home address | Modelled for home address | Urban background | Street level | Measured at home address | Modelled for home address | Urban background | Street level | Measured at home address | Modelled for home address | Urban background | Street level | Measured at home address |  |
| **1 hour average** |  |  |  |  |  |  |  |  |  |  |  |  |  |  |
| Measured at home address | 1 |  |  |  |  |  |  |  |  |  |  |  |  |  |
| Modelled for home address | 0.31 | 1 |  |  |  |  |  |  |  |  |  |  |  |  |
| Measured at continuous monitor: |  |  |  |  |  |  |  |  |  |  |  |  |  |  |
| ·         Urban background | 0.61 | 0.34 | 1 |  |  |  |  |  |  |  |  |  |  |  |
| ·         Street level | 0.31 | 0.31 | 0.48 | 1 |  |  |  |  |  |  |  |  |  |  |
| **24 hours average** |  |  |  |  |  |  |  |  |  |  |  |  |  |  |
| Measured at home address |  |  |  |  | 1 |  |  |  |  |  |  |  |  |  |
| Modelled for home address |  |  |  |  | 0.27 | 1 |  |  |  |  |  |  |  |  |
| Measured at continuous monitor: |  |  |  |  |  |  |  |  |  |  |  |  |  |  |
| ·         Urban background |  |  |  |  | 0.71 | 0.31 | 1 |  |  |  |  |  |  |  |
| ·         Street level |  |  |  |  | 0.37 | 0.56 | 0.47 | 1 |  |  |  |  |  |  |
| **1 week average** |  |  |  |  |  |  |  |  |  |  |  |  |  |  |
| Measured at home address |  |  |  |  |  |  |  |  | 1 |  |  |  |  |  |
| Modelled for home address |  |  |  |  |  |  |  |  | 0.14 | 1 |  |  |  |  |
| Measured at continuous monitor: |  |  |  |  |  |  |  |  |  |  |  |  |  |  |
| ·         Urban background |  |  |  |  |  |  |  |  | 0.42 | -0.004 | 1 |  |  |  |
| ·         Street level |  |  |  |  |  |  |  |  | 0.01 | 0.75 | 0.34 | 1 |  |  |
| **1 year average** |  |  |  |  |  |  |  |  |  |  |  |  |  |  |
| Measured at home address |  |  |  |  |  |  |  |  |  |  |  |  | 1 |  |
| Modelled for home address |  |  |  |  |  |  |  |  |  |  |  |  | 0.70 | 0,63 |


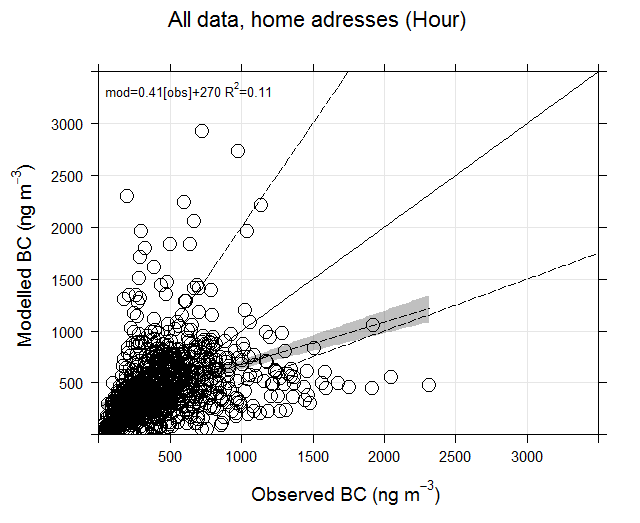

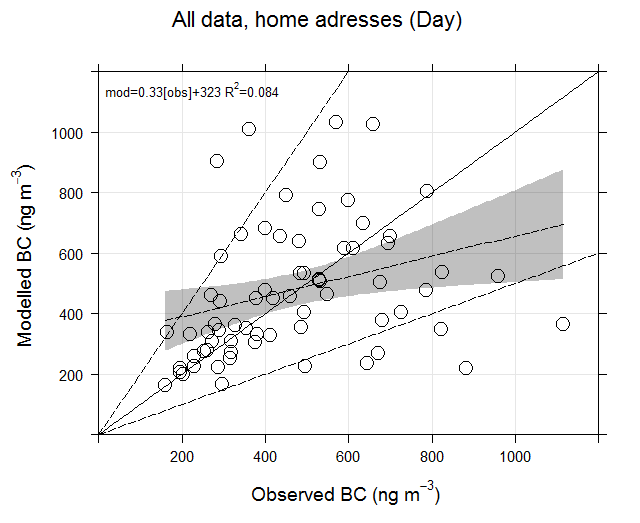

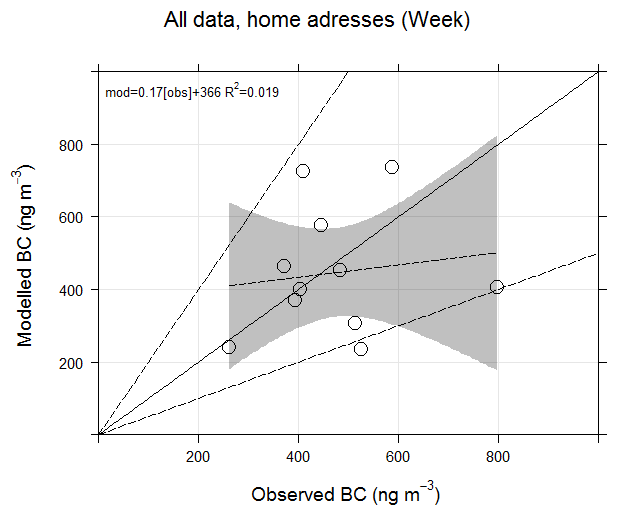

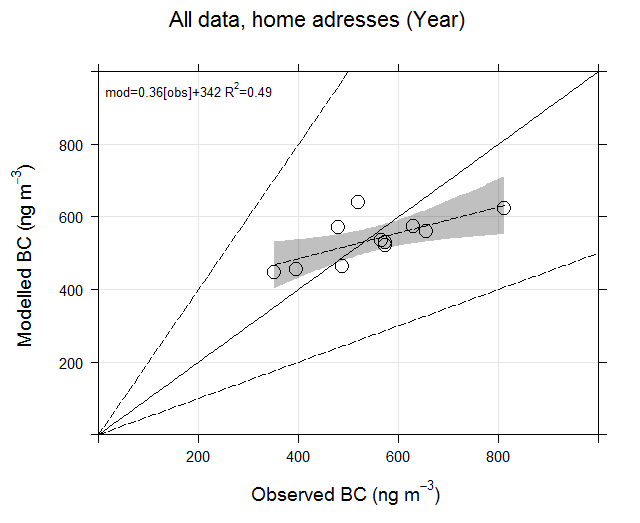


**Fig. S1. Modeled and observed BC hourly, daily and weekly and annual mean concentrations outside the residential homes of 15 families.**

The dashed line is a linear regression and the grey area is the 95 % confidence interval for the linear fit. The solid and dashed lines are 1:1 and 1:2, 1:0.5 lines to show the points that are within a factor of 2 of the observed concentrations.


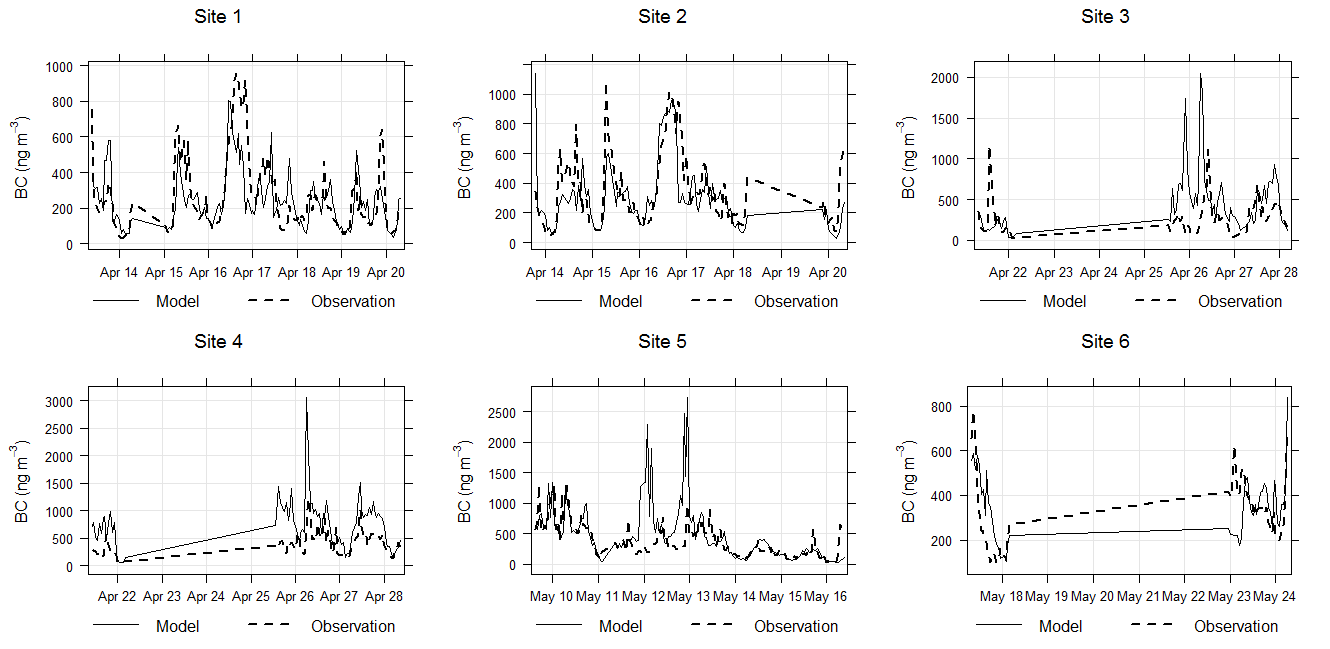

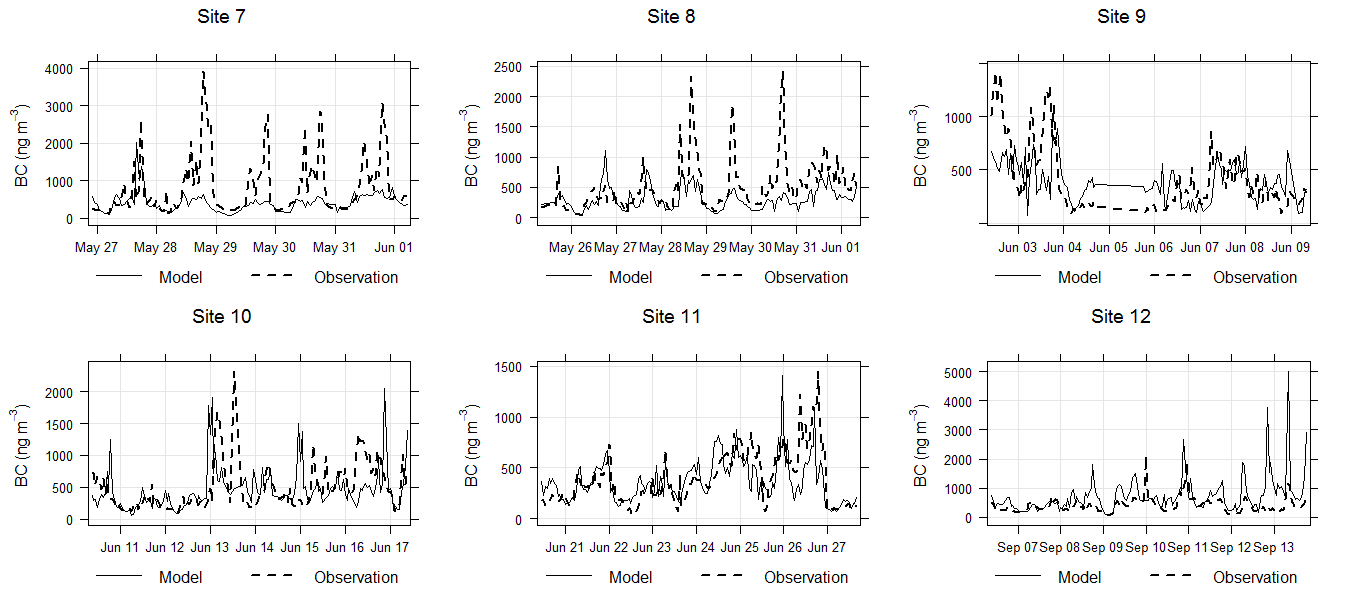


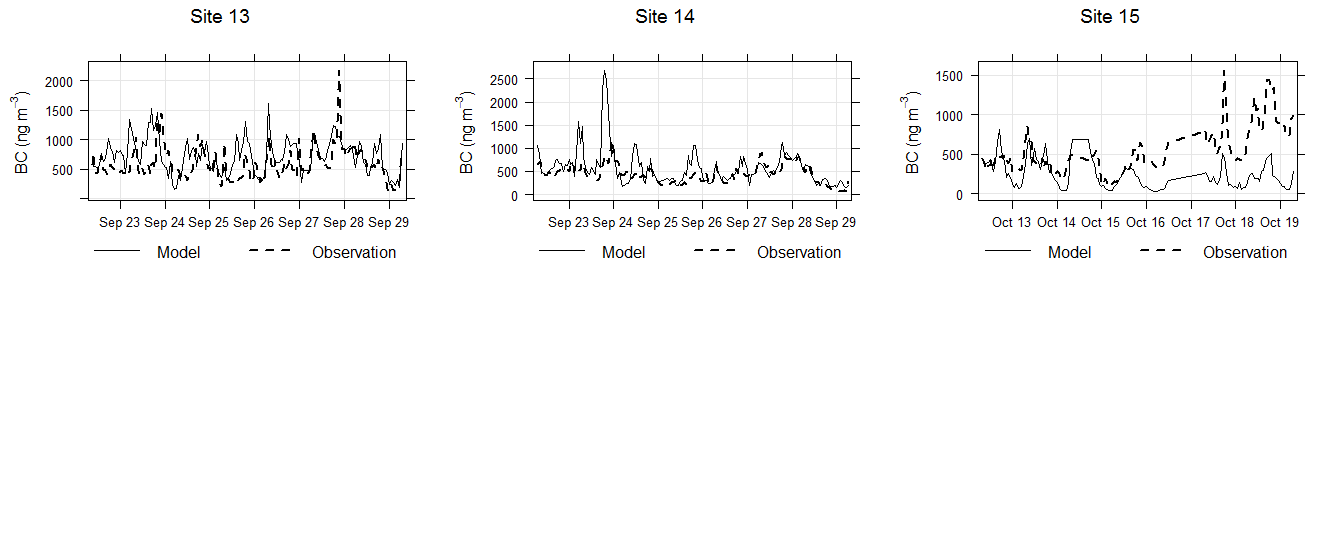


**Fig. S2. Hourly mean modeled (solid lines) and observed (dashed lines) BC concentrations outside the residential homes of 15 families.**
